# Intravitreal methotrexate as a prophylactic strategy for proliferative vitreoretinopathy in high-risk retinal detachment

**DOI:** 10.1186/s12886-026-05455-8

**Published:** 2026-10-17

**Authors:** Adrian Konstantin Luyken, Claudia Brockmann, Valentin Junge, André Schulz, Thomas Armin Fuchsluger, Friederike Schaub

**Affiliations:** Department of Ophthalmology, Rostock University Medical Center, Doberaner Straße. 140, 18057 Hanseatic and University City of Rostock, Germany

**Keywords:** Rhegmatogenous retinal detachment, Proliferative vitreoretinopathy, Methotrexate, Pars plana vitrectomy, Surgical outcomes

## Abstract

**Background:**

Proliferative vitreoretinopathy (PVR) remains the major cause of surgical failure in rhegmatogenous retinal detachment (RRD). This study evaluates the anatomical and functional outcomes, as well as the safety, of a standardized intraoperative intravitreal methotrexate infusion protocol during pars plana vitrectomy (PPV) in eyes with early stage PVR (grade B) or with advanced PVR (grade C).

**Methods:**

A retrospective case series was conducted on 60 consecutive eyes of 59 patients with RRD and PVR grade B (*n* = 29) or grade C (*n* = 31) undergoing PPV with adjunct intraoperative methotrexate between November 2024 and February 2026 at a German tertiary center. Methotrexate 40 mg was administered via a 500 mL balanced salt solution infusion. Functional and structural testing included best-corrected visual acuity (BCVA), intraocular pressure, multifocal electroretinogram, perimetry, and spectral-domain optical coherence tomography. The primary outcome measure was single-surgery anatomical success (SSAS) at 12 weeks after primary surgery or after silicone oil removal. Secondary outcomes included BCVA changes and complication rates.

**Results:**

SSAS was achieved in 81.7% of all eyes, with a significantly higher success rate in eyes with PVR grade B compared to PVR grade C (93.1% versus 71.0%; *p* = 0.043). At 12 weeks, BCVA improved significantly in macula-off cases (*p* < 0.001) and remained stable in macula-on cases. In an exploratory subset of 17 patients postoperative multifocal electroretinogram and perimetry demonstrated preserved central retinal function. No ocular or systemic adverse events related to methotrexate occurred.

**Conclusions:**

The standardized intraoperative intravitreal methotrexate infusion protocol was safe, feasible, and associated with high single-surgery reattachment rates, particularly in PVR grade B eyes. Anatomical outcomes in PVR grade C eyes were comparable to other complex PVR cohorts, without any drug-related local or systemic toxicity.

**Trial registration:**

Not applicable (retrospective observational study).

## Introduction

Rhegmatogenous retinal detachment (RRD) is a vision-threatening condition requiring prompt surgical treatment to prevent irreversible photoreceptor damage and vision loss. Proliferative vitreoretinopathy (PVR) continues to represent the leading cause of surgical failure in retinal detachment repair, with recurrence rates of 10–15% in primary complex cases and over 25% in recurrent cases [1, 2]. Excessive scarring leads to secondary tractional retinal detachments often requiring multiple and complex re-operations to achieve retinal reattachment [1, 3].

Despite substantial advances in vitreoretinal surgical techniques, including small-gauge pars plana vitrectomy (PPV), wide-angle viewing systems, and improved tamponade agents, long-term anatomical and visual outcomes in cases complicated by PVR remain unsatisfactory. There is currently no standard therapy to prevent PVR [4, 5].

PVR is characterized by the formation of contractile, fibrous preretinal and subretinal membranes composed of transdifferentiated retinal pigment epithelial (RPE) cells, glial cells, and inflammatory mediators. These membranes exert traction on the retina, causing re-detachment and distortion of the retinal architecture [6]. The process is thought to represent an excessive wound-healing response involving epithelial-mesenchymal transition (EMT) of RPE and Müller cells, driven by cytokines (e.g., Transforming Growth Factor-beta (TGF-β), Platelet-Derived Growth Factor (PDGF), Tumor Necrosis Factor-alpha (TNF-α) and blood-retinal barrier breakdown [7, 8].

Early vitreous inflammation, driven by T-cell activation and mTOR pathway upregulation (IL-2, IL-6, IL-13), triggers the subsequent proliferation, migration, and EMT of RPE cells and glia [9, 10].

Methotrexate (MTX) inhibits dihydrofolate reductase limiting the DNA synthesis of inflammatory cells [11], reducing early-stage vitreous cytokines (IL-1β, IL-6) and downregulating core EMT markers like ZEB1 and ⍺-SMA [12]. Suppressing these upstream signals halts the proliferation of RPE cells and glia while inducing caspase-3/7 apoptosis at non-toxic concentrations [13, 14].

While intravitreal MTX is increasingly used as an adjunctive surgical therapy to prevent recurrent PVR, its clinical efficacy remains uncertain [4, 15]. Current reactive management with repeated intravitreal MTX injections for established PVR Grade C intervenes after the pathogenic process has already initiated [16, 17]. To target the early inflammatory signaling that causes the subsequent cellular transformation, a prophylactic MTX infusion regimen in early stage PVR (grade A and B) cases may represent a potential therapeutic option to disrupt the disease cascade at its onset, before membrane formation occurs, thereby reducing complex clinical courses and improving long-term anatomical success.

Furthermore, while existing studies have primarily evaluated the safety profile of intravitreal MTX based on clinical complications – such as anterior segment toxicity, corneal epitheliopathy, intraocular pressure elevation, or maculopathy – standardized, objective assessments of retinal function in-vivo remain lacking.

We conducted a retrospective cohort study describing the use of a standardized intraoperative MTX infusion protocol during PPV for RRD in eyes with PVR Grade B or C in a German tertiary referral center. Our primary objective was to characterize the anatomical and functional outcomes, as well as the safety profile, over 12 weeks following primary repair or silicone oil removal. Secondary aims were to explore best-corrected visual acuity (BCVA) changes and complication rates associated with complex RRD surgery and intraoperative MTX infusion. Because this was a single-arm cohort without a non-MTX comparison group, we did not seek to definitively establish MTX efficacy, and all sub-analyses between PVR grades were interpreted descriptively. To the best of our knowledge, this is the first multi-patient cohort to add safety data using multifocal electroretinography (mfERG) as an objective, functional biomarker, focusing on macular safety and tolerability to complement ongoing controlled trials.

## Methods

### Study design

This retrospective, single-center case series was conducted at a German tertiary referral center to evaluate the anatomical outcomes, functional results, and safety profile of an adjunctive intravitreal MTX infusion protocol. The protocol was applied as a prophylactic measure in eyes at high risk for proliferative vitreoretinopathy (PVR Grade B) and as a management strategy in cases with established PVR Grade C during RRD rhegmatogenous repair. The study was approved by the local Institutional Review Board (A 2025-0014) and was conducted in adherence to the tenets of the Declaration of Helsinki. The patients were informed about the off-label use of MTX, and written informed consent was obtained from each patient. In our observation period no patients declined the adjunct therapy.

**Fig. 1:**
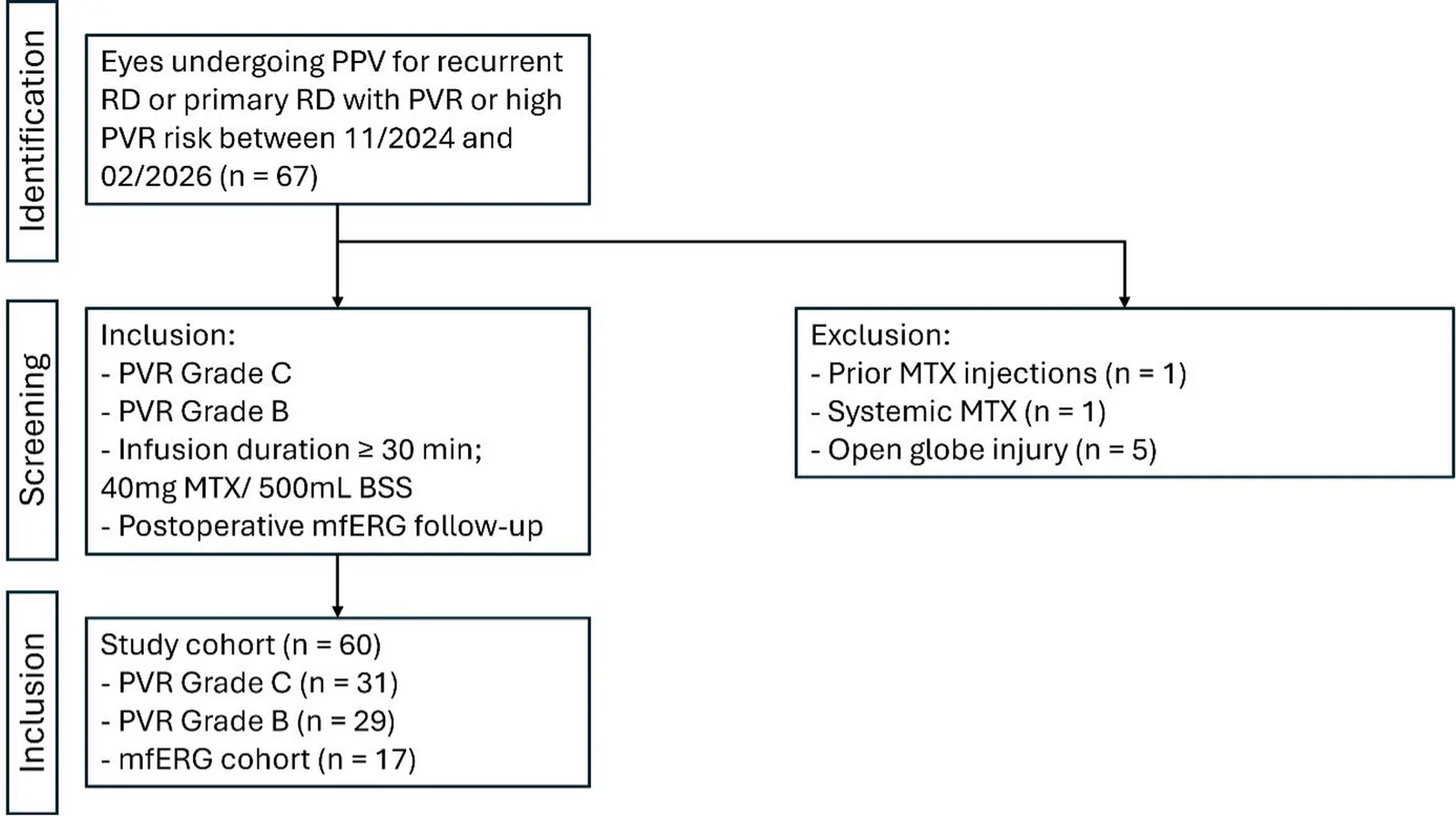
Flowchart of patient selection and retrospective enrollment. Out of 67 eyes initially identified, 7 were excluded based on specific criteria, leaving a final study cohort of 60 eyes (31 with PVR grade C, 29 with PVR grade B, and a subset of 17 in the mfERG cohort). BSS, balanced salt solution; mfERG, multifocal electroretinogram; MTX, methotrexate; PPV, pars plana vitrectomy; PVR, proliferative vitreoretinopathy; RD, retinal detachment

All consecutive patients with RRD complicated by PVR Grade B or C who underwent vitreoretinal reattachment surgery between November 2024 and February 2026 were screened for inclusion (Fig. 1). PVR severity was classified according to the updated criteria of the Retina Society, originally described by Machemer et al. [18]. Exclusion criteria comprised RRD without visible PVR of any grade, tractional diabetic retinal detachment, history of open-globe trauma, active uveitis or ocular infection, and insufficient follow-up data.

### Data collection

Clinical charts were comprehensively reviewed to collect patient demographics, baseline ocular characteristics, surgical parameters, and postoperative outcomes. Demographic data included age and sex. Baseline and baseline-dependent ocular variables comprised lens status (phakic, pseudophakic, or aphakic), operative eye laterality, duration of symptoms prior to surgery (days), total extent of retinal detachment (affected quadrants), and macular status (macula-on versus macula-off). Surgical parameters extracted included the specific intraoperative MTX concentration, total duration of MTX infusion, and the type of intraocular tamponade utilized.

Importantly, no patients were lost to follow-up before the primary 12-week endpoint, resulting in a complete dataset of 60 eyes for the final analysis.

### Surgical technique

All surgical procedures were performed by two experienced vitreoretinal surgeons. Patients underwent standardized small-gauge PPV. In cases presenting with visually significant cataracts, combined phacoemulsification and intraocular lens (IOL) implantation was performed prior to vitrectomy. For intraoperative infusion, 40 mg MTX dissolved in 1 mL of 0.9% sodium chloride, prepared by our in-house pharmacy and stored light-protected at 4 °C for up to 28 days, was injected into a 500mL balanced salt solution (BSS) bottle 5 to 10 min prior to surgery, yielding a final concentration of 80 ug/mL. The infusion bottle was thoroughly mixed following injection, and the infusion line was flushed while avoiding direct contact of the solution with the ocular surface. The infusate was administered under standard ambient operating room lighting without dedicated photoprotection, given its established physicochemical stability over this brief timeframe. The medicated BSS was administered intraoperatively via the infusion line for a minimum of 30 min and up to a maximum of 60 min. If the total surgical time exceeded 60 min, the infusion reservoir was switched back to standard, unmedicated BSS for the remainder of the procedure. Following core vitrectomy and peripheral vitreous shaving under scleral indentation, perfluorocarbon liquid (PFCL) was used, if necessary, all retinal breaks were treated using either endolaser photocoagulation or transconjunctival cryopexy. In cases of advanced PVR (grade C), relaxing retinotomy or retinectomy was carried out as required. Retinal reattachment was subsequently achieved *via* fluid-air exchange. The choice of intraocular endotamponade (Siluron 5000 ^®^, ZEISS, Jena, Germany or 20% sulfur-hexafluoride (SF_6_), Geuder, Heidelberg, Germany) was made on retinal findings with either 20% SF_6_ or light silicone oil (5000 cSt).

Prior to the initiation of this study in November 2024, intraoperative or postoperative MTX was not routinely utilized at our tertiary center. The protocol described herein represents the first systematic implementation of standardized intraoperative MTX infusion at our department.

### Main outcome measures and statistical analysis

Outcome measures were assessed at preoperative baseline, 30 days, 90 days, and the last follow-up. BCVA, intraocular pressure (IOP), anterior chamber status, and fundus findings were evaluated. Spectral-domain optical coherence tomography (SD-OCT) imaging was used to assess macular configuration and detect any signs of postoperative epiretinal membrane formation, cystoid macular edema, or macular hole development. In cases with silicone oil, oil removal was routinely planned at 12 weeks postoperatively, provided the retina was fully attached without active traction or open breaks.

To objectively evaluate macular function and retinal toxicity, patients underwent mfERG (Roland Consult, Brandenburg an der Havel, Germany) and standard automated perimetry (24:2 SITA-Fast, Humphrey Field Analyzer 3, ZEISS Medical Technology, Carl Zeiss AG, Oberkochen, Germany). The rate of re-detachment was evaluated for each eye throughout the entire follow-up period. Time since surgery was included as a continuous variable to capture postoperative recovery trends.

The primary anatomical outcome was single-surgery anatomical success (SSAS), defined as complete retinal reattachment at 12 weeks following primary vitrectomy (in gas-filled eyes) or 12 weeks following silicone oil removal (in oil-filled eyes) without the need for secondary vitrectomy interventions, while secondary metrics included longitudinal re-detachment rates tracked throughout the entire follow-up period, BCVA kinetics modeled continuously, and a comprehensive documentation of all localized or systemic adverse events potentially attributable to MTX toxicity or the surgical procedure itself.

### Statistical analysis

Statistical analyses were performed using Python 3.13.9 with the packages pandas, NumPy, SciPy, statsmodels, and lifelines. Figures were generated with Matplotlib. All package versions are available from the corresponding author upon request. To account for potential intra-patient correlation secondary to the inclusion of both eyes from one bilateral subject, a sensitivity analysis was conducted by restricting the primary outcome evaluations to *n* = 59 independent eyes (randomly excluding one fellow eye). Continuous variables, including BCVA converted to logMAR, were summarized as mean ± standard deviation (SD) and median with interquartile range (IQR). Categorical variables were reported as absolute frequencies and percentages. Proportions were accompanied by Wilson 95% confidence intervals. Mean changes in logMAR BCVA were reported with 95% confidence intervals calculated using the t-distribution. Within-group changes in BCVA compared with baseline were analyzed using two-sided Wilcoxon signed-rank tests. Between-group comparisons, including tamponade type (silicone oil vs. gas) and macular status (macula-on vs. macula-off), were assessed using two-sided Mann-Whitney U tests. Anatomical outcome, including single-surgery success and PVR progression between PVR Grade B and Grade C eyes, were analyzed using two-sided Fisher’s exact test. Time-to-event analysis for recurrent retinal detachment was evaluated using Kaplan–Meier survival curve and compared between PVR Grade B and Grade C using the two-sided log-rank test (Mantel–Cox). The association between infusion duration and risk of re-detachment was assessed using univariable Cox proportional hazards regression and reported as hazard ratio (HR) with 95% confidence interval. All statistical tests were two-sided, with a significance threshold of α = 0.05. Given the exploratory nature of the study, no adjustment for multiple testing was performed.

## Results

### Demographics and clinical characteristics

Sixty eyes from 59 patients (71,2% male [42/59], 55% right eyes [33/60]) with a mean age of 69.6 ± 11.4 years (34–91) received intraoperative MTX infusion. All patients presented with RRD accompanied either by early stage PVR grade B (retinal stiffness, inner surface wrinkling and rolled edges of retinal breaks) or advanced PVR grade C (full-thickness retinal folds) as assessed preoperatively and intraoperatively. At the time of presentation, 45 eyes (75.0%) were classified as macula-off and 15 eyes (25.0%) as macula-on. 29 eyes (48.3%) were graded as PVR grade B, and 31 eyes (51.7%) as PVR grade C, according to the updated Retina Society Classification [18]. The majority of cases (*n* = 47, 78.3%) presented with primary RRD with concomitant PVR, while the remaining (*n* = 13, 21.7%) had a history of prior vitreoretinal surgery due to RRD. At the time of surgery, 34 eyes (56.7%) were pseudophakic, 25 eyes (41.7%) were phakic, and 1 eye (1.7%) was aphakic. Each patient underwent 23-gauge PPV with intraoperative MTX infusion (40 mg MTX diluted in 500 mL [= 80 µg/mL]) via the BSS infusion line. Mean infusion time was 34.4 ± 5.8 min (range 30–60 min). Endotamponade was performed with 20% SF_6_ gas in 21 eyes (35.0%) or light silicone oil (density: 0.97 g/cm³, viscosity: 5000 cSt) in 39 eyes (65.0%). Table 1 summarizes the clinical data.

**Table 1:** Baseline demographics and clinical characteristics (*n* = 60 eyes of 59 patients)

**Demographics**	
Age, years, mean ± SD [range]	69.6 ± 11.4 [33.8–91.5]
Age, years, median [IQR]	70.6 [63.7–76.2]
Male sex, n (%)	42 (71.2)
**Operative eye**,** n (%)**	
Right	33 (55.0)
Left	27 (45.0)
**Diagnosis**,** n (%)**	
Primary retinal detachment	47 (78.3)
Secondary retinal detachment	13 (21.7)
**Preoperative status**,** n (%)**	
Macula-off	45 (75.0)
PVR grade B	29 (48.3)
PVR grade C	31 (51.7)
**Lens status**,** n (%)**	
Phakic	25 (41.7)
Pseudophakic	34 (56.7)
Aphakic	1 (1.7)
Prior vitreoretinal surgery, n (%)	14 (23.3)
**Surgical details**,** n (%)**	
Silicone oil tamponade	39 (65.0)
Gas tamponade	21 (35.0)
Combined phacoemulsification	2 (3.3)
**Preoperative visual acuity**	
logMAR, mean ± SD	1.44 ± 0.88
logMAR, median [IQR]	1.50 [0.67–2.30]
**Follow-up**	
Days, mean ± SD [range]	321.4 ± 142.2 [87–537]
Days, median [IQR]	337.0 [213.2–456.5]

Data are presented as mean ± SD [range], median [IQR], or n (%). Patient-based metrics reflect *n* = 59 patients; eye-based metrics reflect *n* = 60 eyes. BCVA, Best-Corrected Visual Acuity; IQR, Interquartile Range; PVR, Proliferative Vitreoretinopathy; SD, Standard Deviation

The mean follow-up was 321.4 ± 142.2 days (87–537 days). SSAS was achieved in 49 of 60 eyes (81.7%, 95% CI 70.1–89.4%). Among PVR grade B eyes, 27 of 29 (93.1%, 95% CI 78.0–98.1%) achieved single-surgery reattachment (Table 2), compared to 22 of 31 (71.0%, 95% CI 53.4–83.9%) in PVR grade C (*p* = 0.043). 11 eyes (18,3%) required follow-up vitrectomy due to recurrent retinal detachment. Intraoperative retinectomy was performed in 4 cases (6,7%) at the time of re-surgery.

**Table 2:** Anatomical outcomes by initial PVR grade

	Total (*n* = 60)	PVR grade B (*n* = 29)	PVR grade C (*n* = 31)	p*
**Single surgery success**				
n/N (%) [95% CI]	49/60 (81.7) [70.1–89.4]	27/29 (93.1) [78.0–98.1]	22/31 (71.0) [53.4–83.9]	0.043
**Final anatomical success**				
n/N (%) [95% CI]	56/60 (93.3) [84.1–97.4]	29/29 (100) [88.3–100]	27/31 (87.1) [71.1–94.9]	0.116
**PVR progression**				
4–6 weeks, n/N (%)	4/56 (7.1)	1/27 (3.7)	3/29 (10.3)	0.612
12 weeks, n/N (%)	2/36 (5.6)	0/16 (0.0)	2/20 (10.0)	0.492

* Fisher’s exact test; 95% CI by Wilson method. Cox regression: HR = 1.08 per minute of infusion duration (95% CI 1.02–1.15, *p* = 0.01). PVR, Proliferative Vitreoretinopathy

Visual acuity outcome was stratified based on initial macular status (Tables 3 and 4). In the macula-off group (*n* = 45, 75%), mean BCVA improved significantly from 1.81 ± 0.67 logMAR (range 0.20–2.70) preoperatively to 0.68 ± 0.42 logMAR (range 0.10–2.00) at 12 weeks post-surgery (*p* < 0.001, Wilcoxon signed-rank test). In eyes that presented with macula-on status (*n* = 15, 25%), BCVA remained stable, showing a minor, statistically non-significant change from 0.31 ± 0.25 logMAR (range 0.00–0.92) to 0.44 ± 0.34 logMAR (range 0.20–1.30); *p* = 0.469).

**Table 3:** Best-corrected visual acuity outcomes: logMAR mean (SD)

	Total (*n* = 60)	Silicone oil (*n* = 39)	Gas (*n* = 21)	Macula-on (*n* = 15)	Macula-off (*n* = 45)
Baseline BCVA	1.44 (0.88)	1.49 (0.86)	1.35 (0.92)	0.31 (0.25)	1.81 (0.67)
4–6 weeks	0.56 (0.43)	0.68 (0.41)	0.35 (0.41)	0.37 (0.28)	0.62 (0.46)
Δfrom baseline	0.86 (0.79)	0.79 (0.76)	1.00 (0.84)	−0.06 (0.24)	1.18 (0.65)
12 weeks	0.51 (0.39)	0.65 (0.31)	0.28 (0.41)	0.35 (0.31)	0.57 (0.40)
Δfrom baseline	0.90 (0.80)	0.80 (0.77)	1.06 (0.85)	−0.04 (0.25)	1.23 (0.65)

Best-corrected visual acuity (BCVA) values are displayed in logMAR mean (SD). Wilcoxon signed-rank test for within-group changes; Mann–Whitney U test for between-group comparisons. Δ values are based on paired observations; three patients in the silicone oil group had no postoperative visual acuity documented (*n* = 1 at 4–6 weeks, *n* = 2 at 12 weeks) and were excluded from the respective Δ calculations

**Table 4:** Best-corrected visual acuity: subgroup comparisons (logMAR mean [SD])

	Silicone oil (*n* = 39)	Gas (*n* = 21)	p*	Macula-on (*n* = 15)	Macula-off (*n* = 45)	p*
Baseline BCVA	1.49 (0.86)	1.35 (0.92)	0.742	0.31 (0.25)	1.81 (0.67)	< 0.001
4–6 weeks	0.68 (0.41)	0.36 (0.42)	< 0.001	0.39 (0.28)	0.63 (0.47)	0.091
Δfrom baseline	0.76 (0.75)	1.00 (0.84)	0.229	−0.07 (0.25)	1.15 (0.65)	< 0.001
95% CI of Δ	[0.51; 1.01]	[0.60; 1.41]		[− 0.21; 0.07]	[0.95; 1.35]	
p vs. baseline	< 0.001	< 0.001		0.449	< 0.001	
12 weeks	0.68 (0.32)	0.43 (0.60)	0.004	0.44 (0.34)	0.68 (0.42)	0.077
Δfrom baseline	0.71 (0.72)	1.19 (0.85)	0.149	−0.10 (0.30)	1.14 (0.61)	< 0.001
95% CI of Δ	[0.43; 0.99]	[0.54; 1.85]		[− 0.33; 0.13]	[0.90; 1.38]	
p vs. baseline	< 0.001	0.008		0.469	< 0.001	

* Mann–Whitney U test for between-group comparisons (silicone oil vs. gas; macula-on vs. macula-off). Wilcoxon signed-rank test for within-group p vs. baseline. Δ values are based on paired observations; three patients in the silicone oil group had no postoperative visual acuity documented (*n* = 1 at 4–6 weeks, *n* = 2 at 12 weeks) and were excluded from the respective Δ calculations

### Functional and structural outcomes

**Fig. 2:**
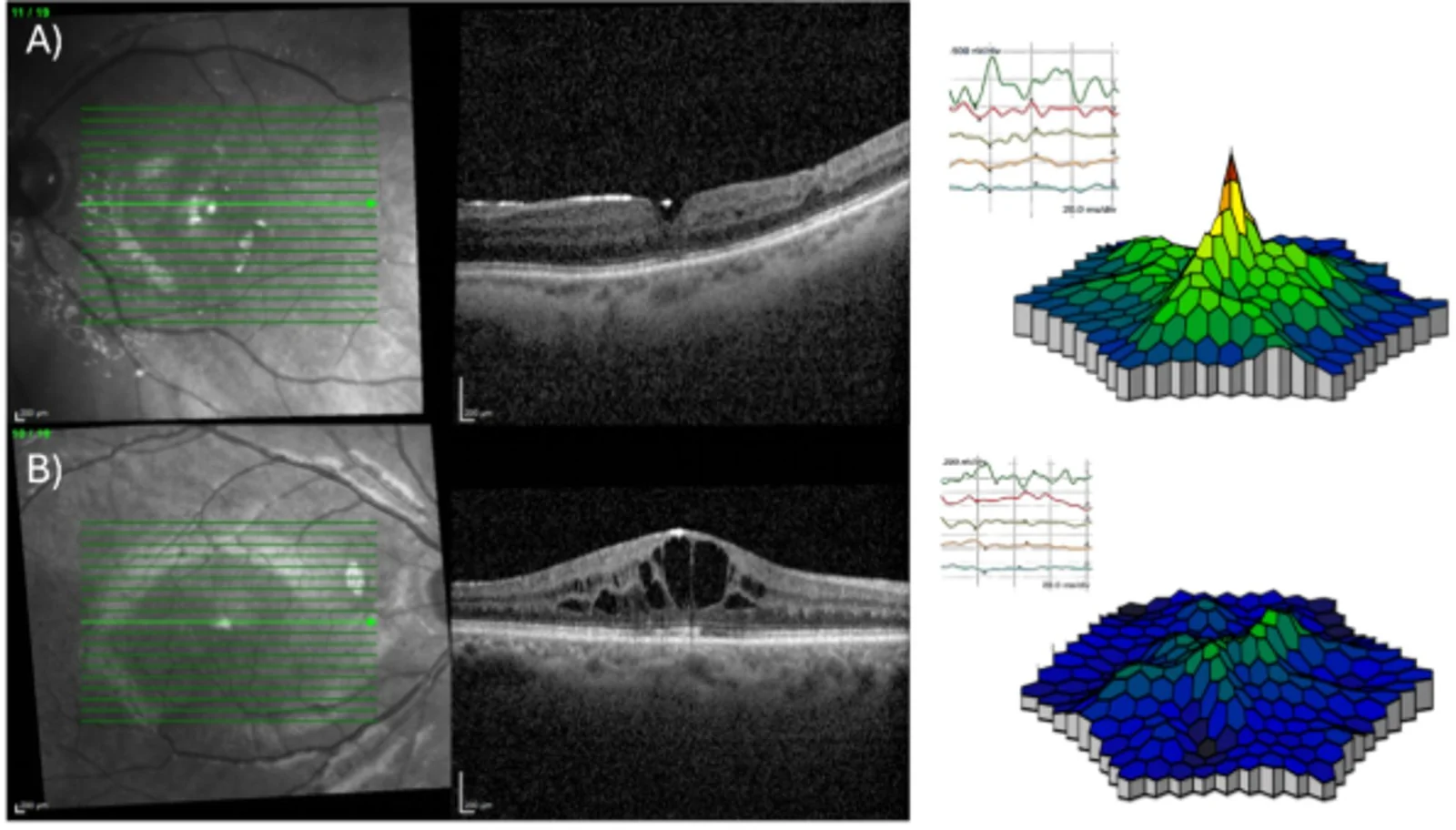
**a**) Macular OCT and mfERG of an 82-year-old patient 12 weeks after PPV with MTX for PVR retinal detachment with a normal macula and unremarkable foveal response. OCT, Ocular Coherence Tomography; mfERG, Multifocal Electroretinogram; PPV, Pars Plana Vitrectomy; MTX, Methotrexate. **b**) Macular OCT and multifocal electroretinogram of an 87-year-old patient 12 weeks after PPV with MTX for PVR detachment with cystoid macular edema and flattened foveal peak under silicone oil tamponade. OCT, Optical Coherence Tomography; mfERG, Multifocal Electroretinogram; PPV, Pars Plana Vitrectomy; MTX, Methotrexate

A subset of 17 patients underwent mfERG and standard automated perimetry at the 3-month follow-up visit. In eyes presenting with baseline macula-on status and complete morphological resolution of macular edema, foveal peak responses and topographical response amplitudes remained within physiological limits (Fig. 2a). Conversely, reduced response amplitudes and alterations in implicit times were observed in eyes with a history of baseline macula-off retinal detachment or persisting intraretinal fluid at 12 weeks (Fig. 2b). Morphological analysis *via* SD-OCT confirmed the restoration of a regular foveal contour in successfully reattached macula-off cases without persistent fluid, while no cases with, subfoveal fibrosis or full-thickness macular holes were observed across the evaluated subgroup.

### Safety profile and postoperative complications

Postoperative complications were recorded in 22 of 60 patients (36.7%), with no documented intraocular, ocular surface, or systemic events attributed to MTX-specific toxicity (Table 5). Retinal re-detachment occurred in 11 cases (18.3%), and PVR progression was identified in 5 patients (8.3%). Macular edema was detected via OCT at various postoperative timepoints in 12 patients (20.0%), presenting in 10 patients (16.7%) at 4–6 weeks and in 5 patients (8.3%) at 12 weeks, and in one case with persistent macular edema (ME) even after intravitreal triamcinolone acetonide injection during concurrent silicone oil removal and re-operation. Elevation of IOP to 25 mmHg or higher was observed in 3 patients (5.0%), which was successfully managed with temporary topical IOP-lowering therapy. Accelerated cataract formation seen at 12 weeks postop occurred in 4 patients (6.7%), both tracking within expected rates for complex vitreoretinal interventions. Residual subretinal perfluorcarbon liquid (PFCL) was noted in 1 patient (1.7%). No patient presenting with baseline macula-on status experienced unexpected, unexplained, or permanent visual acuity loss during follow-up.

**Table 5:** Postoperative complications (*n* = 60)

**Any complication**	22/60 (36.7)
Re-detachment	11/60 (18.3)
Macular edema (at any timepoint)	12/60 (20.0)
at 4–6 weeks	10/60 (16.7)
at 12 weeks	5/60 (8.3)
PVR progression (at any timepoint)	5/60 (8.3)
Cataract (out of 25 phakic eyes)	4/25 (16.0)
IOP elevation (≥ 25 mmHg)	3/60 (5.0)
Residual subretinal PFCL	1/60 (1.7)
MTX-related toxicity	0/60 (0)

Values are n/N (%). Macular edema data from postoperative OCT records. No cases of corneal toxicity or other MTX-related adverse events were documented. PVR, Proliferative Vitreoretinopathy; IOP, Intraocular Pressure; MTX, Methotrexate

Regarding the safety profile of the intraoperative MTX infusion protocol, no clinical signs of corneal epithelial toxicity, persistent keratopathy, or prolonged anterior chamber inflammation were observed throughout the study period. The low rate of IOP elevation and the absence of superficial punctate keratitis or other adverse ocular surface events contrast with the complication rates documented in serial intravitreal injection regimens. Furthermore, no systemic discomfort or medication-related adverse side effects were reported by any patient in this cohort.

### Endotamponade selection

The gas tamponade group achieved a significantly higher single-surgery success rate compared to the silicone oil group (100% vs. 71.8%; *p* = 0.006) and experienced no recurrent retinal detachments (0% vs. 23.1%; *p* = 0.021) (Table 6).

**Table 6:** Outcomes by tamponade type

	Silicone oil (*n* = 39)	Gas (*n* = 21)	p*
**Anatomical outcomes**			
Single surgery success, n/N (%) [95% CI]	28/39 (71.8%) [56.2–83.5%]	21/21 (100.0%) [84.5–100.0%]	0.006
Re-detachment, n/N (%) [95% CI]	9/39 (23.1%) [12.6–38.3%]	0/21 (0.0%) [0.0–15.5%]	0.021
PVR progression (any), n/N (%)	5/39 (12.8%)	0/21 (0.0%)	0.152
Macular edema (any), n/N (%)	10/39 (25.6%)	2/21 (9.5%)	0.185

* Fisher’s exact test for categorical outcomes; 95% CI by Wilson method. PVR, Proliferative Vitreoretinopathy

### Lens status

No statistically significant differences in primary anatomical outcomes were observed between phakic and pseudophakic eyes. Two specific parameters neared but did not reach statistical significance: progressive PVR was documented in 14.7% of pseudophakic eyes compared to 0% of phakic eyes (*p* = 0.066), and postoperative macular edema occurred in 29.4% of pseudophakic eyes versus 8.0% of phakic eyes (*p* = 0.055) (Table 7).

**Table 7:** Outcomes by lens status (*n* = 59; one aphakic eye excluded from analysis)

	Phakic (*n* = 25)	Pseudophakic (*n* = 34)	p*
**Anatomical outcomes**			
Single surgery success, n/N (%) [95% CI]	22/25 (88.0%) [70.0–95.8%]	27/34 (79.4%) [63.2–89.7%]	0.494
Re-detachment, n/N (%) [95% CI]	2/25 (8.0%) [2.2–25.0%]	6/34 (17.6%) [8.3–33.5%]	0.447
PVR progression (any), n/N (%)	0/25 (0.0%)	5/34 (14.7%)	0.066
Macular edema (any), n/N (%)	2/25 (8.0%)	10/34 (29.4%)	0.055

* Fisher’s exact test for categorical outcomes; 95% CI by Wilson method. Aphakic (*n* = 1) excluded from all comparisons. PVR, Proliferative Vitreoretinopathy

## Discussion

In this retrospective case series, a standardized intraoperative MTX infusion protocol was used during PPV for RRD complicated by PVR Grade B or C. Most eyes achieved anatomical stabilization, although structural and functional outcomes differed according to baseline PVR severity. Given the descriptive design and absence of a control group, the findings primarily support the feasibility and local tolerability of this approach rather than a definitive therapeutic effect.

### Influence of baseline lens status and tamponade type

Lens status and endotamponade selection are linked indicators of baseline severity in RD with PVR. Although pseudophakia and aphakia correlate with increased PVR progression due to the loss of the lens barrier [19, 20], the absence of the lens can improve visualization for vitreous base dissection and membrane peeling, yielding higher reattachment rates than phakic configurations [21].

Regarding tamponades, gas and standard silicone oil demonstrate equivalent long-term anatomical success in standard PVR [19], though gas preserves superior functional outcomes by avoiding oil-induced retinal thinning [22]. In severe grade C PVR requiring inferior retinectomy, standard protocols utilize lensectomy and silicone oil to counter mechanical traction [23]; heavy silicone oil shows no advantage over standard oil combined with scleral buckling [24]. Consequently, neither lens status nor endotamponade choice represents an independent variable; both serve as clinical surrogates for baseline disease severity that dictate the surgical approach.

### Functional outcomes

Functional outcomes at 12 weeks correlated with baseline macular status and postoperative morphology rather than drug-related effects. Localized reductions in amplitude and delayed implicit times observed *via* mfERG correspond directly to baseline macula-off status or persistent intraretinal fluid. These alterations align with established literature on functional recovery following complex retinal detachment surgery [25, 26]. These reduced electrophysiological responses correspond to the cumulative neuroretinal damage associated with chronic neurosensory detachment, prolonged macular distortion, and the mechanical stress of surgical membrane peeling. Similar functional baseline variations and limitations have been described in historical cohorts managed with multi-dose serial injection protocols [17].

### PVR severity and anatomical outcomes

**Fig. 3:**
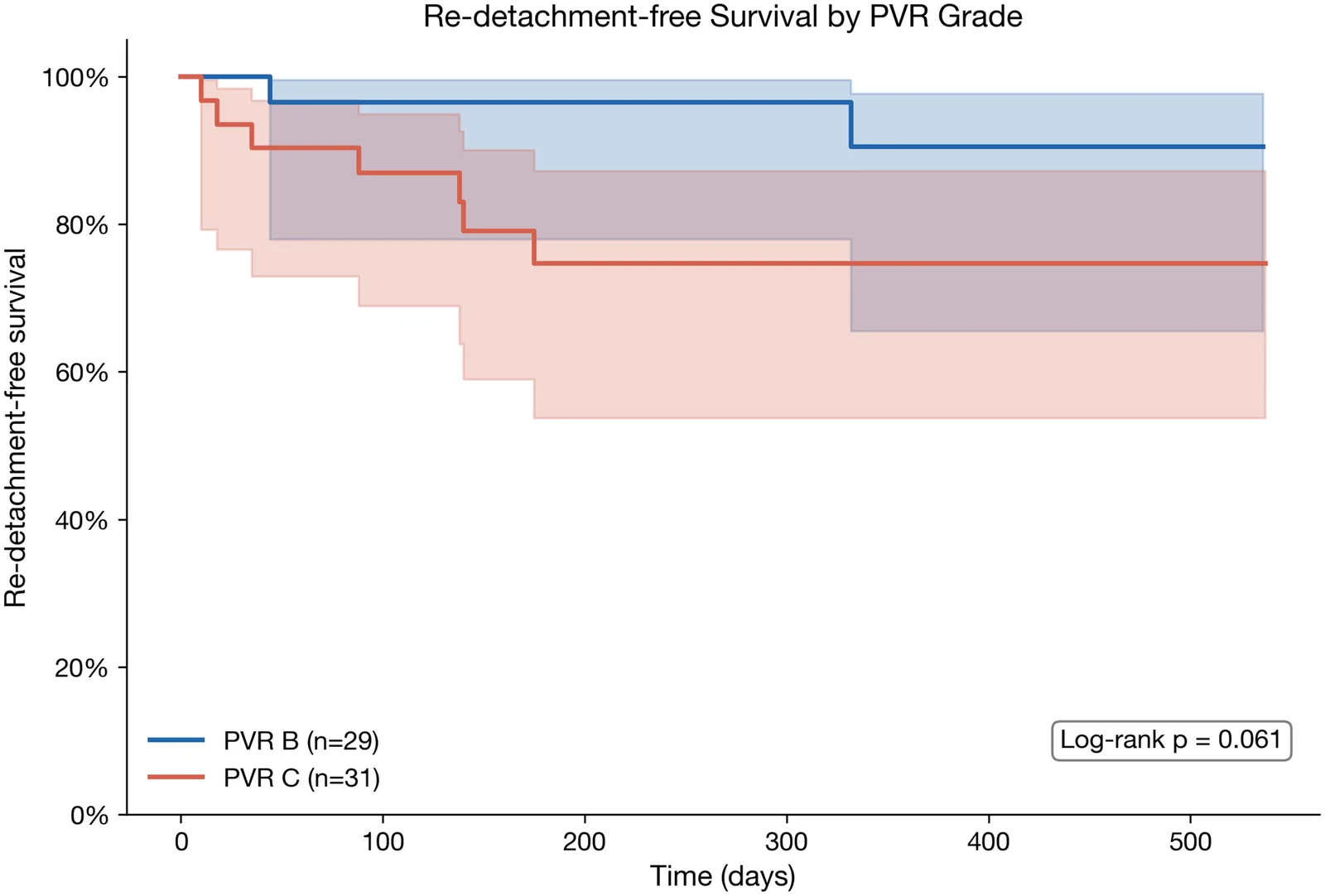
Kaplan–Meier analysis of re-detachment-free survival stratified by preoperative PVR grade. Patients with PVR grade B (*n* = 29, 2 events) demonstrated superior re-detachment-free survival compared to those with PVR grade C (*n* = 31, 7 events), with an early clustering of events in the grade C group during the first 200 postoperative days. The difference did not reach statistical significance (log-rank *p* = 0.061). Shaded areas represent 95% confidence intervals. Median follow-up was 337 days (IQR 213–456). PVR, proliferative vitreoretinopathy

**Fig. 4:**
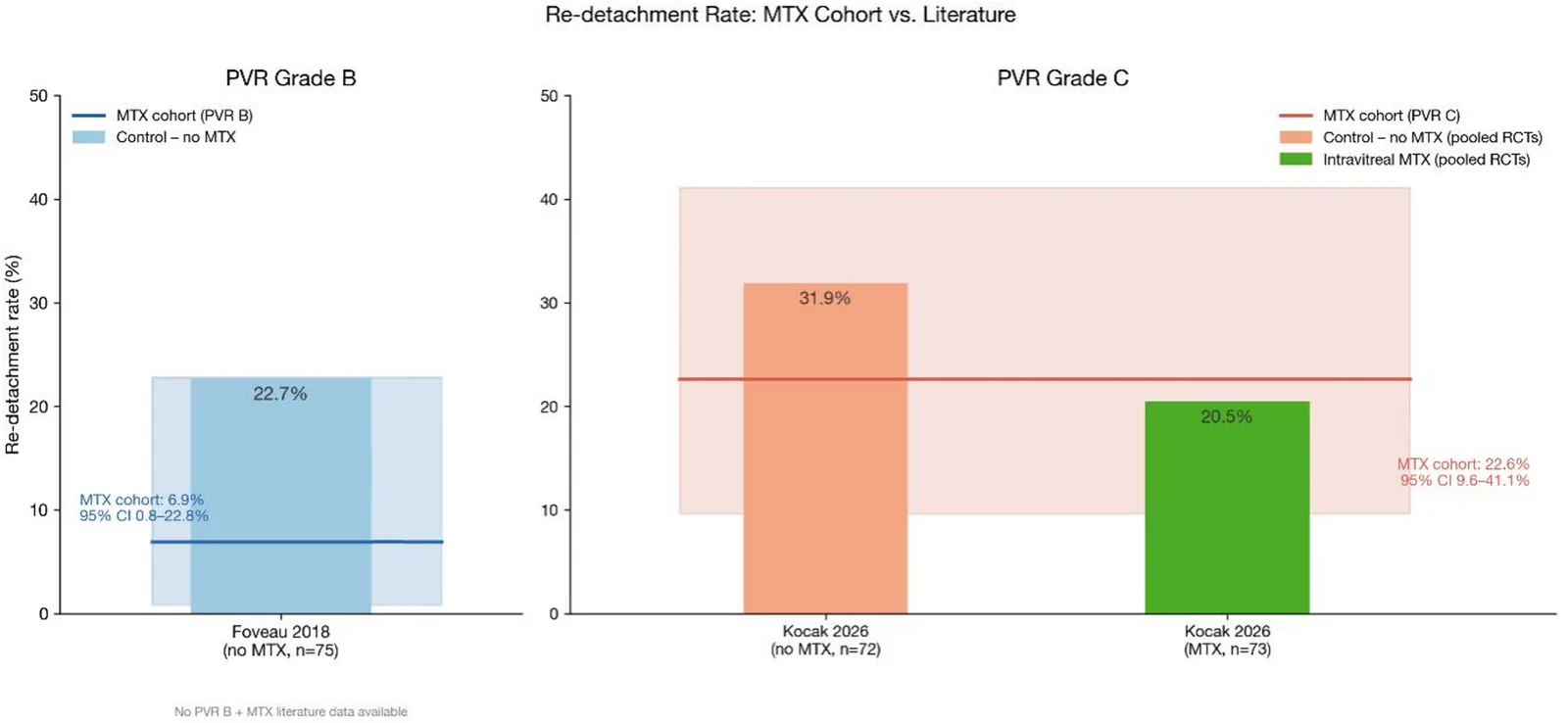
Re-detachment rates of the MTX cohort compared to published literature, stratified by PVR grade. Left panel (PVR Grade B): Our cohort re-detachment rate (6.9%; 95% CI 0.8–22.8%) vs. the control rate reported by Foveau et al. 2018 (22.7%). No MTX-treated comparison series exists for PVR Grade B. Right panel (PVR Grade C): Our cohort re-detachment rate (22.6%; 95% CI 9.6–41.1%) vs. either the pooled control arm (31.9%) or the pooled intravitreal MTX arm (20.5%) of the meta-analysis by Kocak et al. 2026 (3 RCTs). Bars = literature benchmark rates. Horizontal line = MTX cohort rate. Shaded band = 95% CI (Wilson method). CI, confidence interval; MTX, methotrexate; PVR, proliferative vitreoretinopathy; RCT, randomized controlled trial

Eyes with baseline PVR Grade C showed substantially less favorable outcomes than Grade B eyes (Fig. 3). Cox regression suggested an increased risk of surgical failure in Grade C disease (HR 4.05, 95% CI 0.83–19.64, *p* = 0.08), although statistical power was limited. Reported single-surgery reattachment rates in studies using serial intravitreal MTX injections for established PVR Grade C range from 74% to 88% [15, 27, 28]. Anatomical outcomes in our PVR Grade C cohort were within a comparable range despite the use of a single intraoperative administration strategy (Fig. 4). However, direct comparison remains difficult because of heterogeneity in study design, PVR extend, surgical technique, surgeon expertise, tamponade use, and follow-up duration.

Although PVR grade B eyes demonstrated high anatomical stability with limited progression toward advanced tractional disease, the superior outcomes are heavily confounded by baseline disease severity and surgical complexity, rather than MTX action alone. Literature addressing isolated PVR grade B cohorts remains scarce, particularly regarding progression rates to advanced PVR [1, 29]. These preliminary findings suggest that early intraoperative MTX exposure may be most relevant during the initial proliferative phase, before mature fibrotic membrane formation occurs.

### Mechanistic considerations

Various protocols for intravitreal MTX administration in PVR have been described, ranging from serial postoperative injections to single intraoperative applications. We specifically chose a standardized intraoperative infusion protocol over serial postoperative injections for several key reasons. First, PVR-inducing cellular cascades are triggered immediately by the surgical trauma itself; delivering MTX during PPV targets these pathways at the earliest possible stage. Second, hydrophilic MTX injected into eyes filled with silicone oil accumulates in the remaining aqueous phase thus resulting in much higher, possibly toxic concentrations at the retina. Altered pharmacokinetic dynamics and dependency on postoperative head tilt drastically reduce comparability. Third, the infusion approach significantly reduces the therapeutic and psychological burden on patients, avoiding the cumulative risks of endophthalmitis, transient intraocular pressure spikes, and corneal epitheliopathy associated with serial postoperative injections.

The biological rationale for this timing is supported by current understanding of PVR pathogenesis. Retinal detachment and surgical trauma induce breakdown of the blood-retinal barrier with subsequent cytokine release, cellular proliferation, and EMT of RPE and Müller cells. MTX may interfere with these early proliferative processes through inhibition of DNA synthesis and suppression of profibrotic signaling pathways. Experimental studies have demonstrated reduced RPE proliferation and migration following MTX exposure [13, 14]. However, once dense collagenous membranes are established, anti-proliferative therapy alone is unlikely to fully counteract tractional remodeling, which may explain the less favorable outcomes observed in advanced PVR.

### Comparison with previous literature

Our findings align with previous reports evaluating MTX as an adjunctive therapy in PVR. Sadaka et al. first described intraoperative MTX infusion in eyes with high risk for PVR, reporting anatomical stabilization [30], and subsequent studies [31, 32] summarized systematic reviews reported reattachment rates between 74% and 92% across various MTX protocols [17]. The meta-analysis by Kocak et al., which pooled three randomized trials, found a reduction in recurrent retinal detachment (RR 0.64, 95% CI 0.30–1.38) that did not reach statistical significance, which the authors attributed to limited statistical power and methodological heterogeneity across available studies [15].

Stratification by baseline PVR grade in our cohort showed a difference in outcomes between the two subgroups (Fig. 3). In the PVR Grade B subgroup, the re-detachment rate was 6.9% (2/29; 95% CI 0.8–22.8%). This rate is significantly lower than the 22.7% reported by Foveau et al. [33] in a cohort of macula-off detachments with PVR Grade B managed without adjuvant therapy (*n* = 75), (Fig. 4). This finding is particularly unique because no MTX-treated comparison series currently exists in the literature for PVR Grade B.

In the PVR Grade C subgroup, our re-detachment rate was 22.6% (7/31; 95% CI 9.6–41.1%). To put this into a broader clinical context, we compared this outcome to the recent meta-analysis by Kocak et al. which pooled data from three randomized controlled trials [15]. Our rate aligns with either the pooled untreated control arm, which stood at 31.9%, or the pooled intravitreal MTX arm, which was 20.5%. This highlights that while our intraoperative infusion protocol delivers outcomes comparable to intensive postoperative MTX injection regimens for advanced PVR grade C, its most pronounced and novel prophylactic value lies in early-stage intervention during PVR grade B.

Taken together, these data point to a grade-dependent outcome profile for intraoperative MTX, with lower re-detachment rates relative to historical data observed at the PVR grade B level. Clear determination of efficacy across different PVR stages requires randomized trials with prospective grade stratification and larger sample sizes.

### Safety and pharmacokinetic considerations

Although, endothelial cell counts were not routinely performed, an important observation in our cohort was the absence of clinically significant corneal toxicity. Serial intravitreal injection protocols have been associated with cumulative ocular surface complications, including corneal epitheliopathy and keratitis, particularly in studies requiring repeated postoperative injections [34–36]. The closed intraoperative infusion system may reduce localized anterior segment exposure and avoid repeated injection. The observed rate of postoperative macular edema in our series aligns with previously reported incidence rates in complex retinal detachment cases [37, 38], providing no indication of a causal association with intraoperative methotrexate infusion. Mean IOP remained stable throughout follow-up, and no patient required glaucoma surgery. Although these findings support local tolerability, longer follow-up is necessary to exclude delayed toxicity. Pharmacokinetic interpretation remains limited. Available data are largely derived from rabbit models, which demonstrate relatively short intravitreal MTX half-lives [39, 40]. Extrapolation to vitrectomized human eyes is difficult because vitreous removal and silicone oil tamponade substantially alter intraocular fluid dynamics and drug clearance [41]. The effective intraocular exposure achieved with intraoperative infusion therefore remains uncertain.

### Limitations

The study has several limitations. Its retrospective design introduces potential selection bias, as treatment decisions were based on surgeon judgment rather than standardized enrollment criteria. The absence of a parallel control group prevents causal interpretation of anatomical or functional outcomes or any definitive conclusions regarding therapeutic efficacy. Statistical power was further limited by the relatively small number of recurrent detachments, resulting in wide confidence intervals. In addition, follow-up may not capture delayed re-detachment or long-term retinal toxicity. Furthermore, postoperative intraretinal fluid and cystoid macular edema as sign of toxicity could not be reliably distinguished from expected postoperative inflammatory changes after complex retinal detachment repair. Additionally, as a tertiary referral center specializing in maximum care, our patient cohort may not be completely balanced, as predominantly complex and severe cases are referred to our department for surgical management. Finally, because many patients undergo routine postoperative follow-up care closer to their home areas, long-term tracking within our institution was partially restricted.

## Conclusion

In summary, intraoperative MTX infusion during PPV for RRD with PVR grade B or C appeared feasible and locally well tolerated in this cohort. Outcomes differed substantially according to baseline PVR severity, with more favorable structural recovery observed in earlier-stage disease. While these findings support further investigation of intraoperative MTX as a prophylactic adjunct, the current data remain hypothesis-generating. Prospective randomized studies are required to define its therapeutic value, optimal dosing strategy, and appropriate patient selection.

## Data Availability

The datasets used and/or analyzed during the current study are available from the corresponding author on reasonable request.
